# Eyestalk transcriptome and methyl farnesoate titers provide insight into the physiological changes in the male snow crab, *Chionoecetes opilio*, after its terminal molt

**DOI:** 10.1038/s41598-023-34159-y

**Published:** 2023-05-03

**Authors:** Kenji Toyota, Takeo Yamamoto, Tomoko Mori, Miyuki Mekuchi, Shinichi Miyagawa, Masaru Ihara, Shuji Shigenobu, Tsuyoshi Ohira

**Affiliations:** 1 Noto Marine Laboratory, Institute of Nature and Environmental Technology, Kanazawa University, Ogi, Noto-cho, Ishikawa, 927-0553 Japan; 2 Department of Biological Science and Technology, Faculty of Advanced Engineering, Tokyo University of Science, 6-3-1 Niijuku, Katsushika-ku, Tokyo, 125-8585 Japan; 3 Department of Biological Sciences, Faculty of Science, Kanagawa University, 2946 Tsuchiya, Hiratsuka, Kanagawa 259-1293 Japan; 4 Miyazu Field Station, Fisheries Technology Institute, Japan Fisheries Research and Education Agency, 1721 Odasyukuno, Miyazu, Kyoto 626-0052 Japan; 5 Trans-Omics Facility, National Institute for Basic Biology, Okazaki, 444-8585 Japan; 6 Yokohama Field Station, Fisheries Resources Institute, Japan Fisheries Research and Education Agency, 2-12-4 Hukuura, Kanazawa-ku, Yokohama, Kanagawa 236-8648 Japan; 7 Faculty of Agriculture and Marine Science, Kochi University, 200 Monobe-Otsu, Nankoku, Kochi 783-8502 Japan

**Keywords:** Animal physiology, Molecular ecology

## Abstract

The snow crab, *Chionoecetes opilio*, is a giant deep-sea brachyuran. While several decapod crustaceans generally continue to molt and grow throughout their lifetime, the snow crab has a fixed number of molts. Adolescent males continue to molt proportionately to their previous size until the terminal molt at which time an allometric increase in chela size occurs and an alteration of behavioral activities occurs, ensuring breeding success. In this study, we investigated the circulating concentrations of methyl farnesoate (an innate juvenile hormone in decapods) (MF) before or after the terminal molt in males. We then conducted eyestalk RNAseq to obtain molecular insight into the regulation of physiological changes after the terminal molt. Our analyses revealed an increase in MF titers after the terminal molt. This MF surge may be caused by suppression of the genes that encode MF-degrading enzymes and mandibular organ-inhibiting hormone that negatively regulates MF biosynthesis. Moreover, our data suggests that behavioral changes after the terminal molt may be driven by the activation of biogenic amine-related pathways. These results are important not only for elucidating the physiological functions of MFs in decapod crustaceans, which are still largely unknown, but also for understanding the reproductive biology of the snow crab.

## Introduction

In Arthropod species, molting is an indispensable biological process. Successful molting plays a key role in regulating survival, development, metamorphosis, and reproduction^1^. While several decapod crustaceans generally continue to molt and grow throughout their lives, several species such as spider crabs (Majidae) and snow crabs (Oregoniidae) have a fixed number of molts in their lifetime and undergo an allometric differentiation of their chelae after the terminal molt^2^. Crabs that have undergone a terminal molt are unable to undergo additional molting. It is widely accepted that the molting process is centrally regulated by ecdysteroids (the active form is a 20-hydroxyecdysone: 20E) in arthropods, including decapod species^1^. In decapod species, ecdysteroids are synthesized in and released from the Y-organ. Previous studies reported that the Y-organ may shrink or degenerate to inhibit the terminal molt^3–5^. Indeed, circulating ecdysteroid levels are higher in young crabs than in sexually-matured males^5–7^. With regard to the terminal molt, another endocrine factor, juvenile hormone, has been suggested to be relevant in addition to the ecdysteroids. In particular, methyl farnesoate (MF), a juvenile hormone, is a putative innate molecule in decapod species. Several studies have found that the large-carapace abraded large-claw males are the primary reproductive individuals with high circulating and synthetic rates of MF in the spider crab *Libinia emarginata*^4,8^. In particular, high levels of MF were positively correlated with high reproductive behavior^8,9^. Additionally, in snow crabs, *Chionoecetes opilio*, circulating MF levels differed not only before and after the terminal molt, but also depending on the time that elapsed after each molt, which is classified as a "new-shell", in which there is a newly hardened and clean carapace, and the "old-shell", which is characterized by abrasions and epibiotic growth on the carapace^10^. That study divided male snow crabs into four categories: (1) before the terminal molt of the new-shell, (2) before the terminal molt of the old-shell, (3) after the terminal molt of the new-shell, and (4) after the final molt of the old-shell, and demonstrated that highest peak of MF was detected in (1) before the terminal molt of the new-shell while lowest levels of MF were detected in both old-shell groups^10^. Consistent with the comparison of MF levels between new-shell and old-shell before and after the terminal molt is that the new-shell group tends to have higher MF concentrations than the old-shell, indicating the importance of considering the time that has elapsed after molting when measuring MF levels in the snow crab. Although the correlation between the terminal molt and endogenous MF titers varies among crab species, the common feature is a clear increase/decrease in MF levels before or after the terminal molt. This may indicate that rapid changes in MF titer are important for morphological and reproductive behavioral changes after the terminal molt, although its mechanism is unknown.

In decapod crustaceans, MF is synthesized by and secreted from the mandibular organ (MO)^10–12^. Synthesis of MF in the MO is negatively regulated by mandibular organ-inhibiting hormone (MOIH), which was isolated from *L. emarginata* and the brawn crab *Cancer pagurus*^13,14^. MOIH is produced in and secreted from the X-organ/sinus gland (XO/SG) complex located in the eyestalk. The XO/SG complex regulates the biosynthesis and secretion of a wide variety of neuropeptides as well as MOIH. Crustacean hyperglycemic hormone (CHH) is a polypeptide hormone that was originally identified in the XO/SG complex, and its CHH superfamily comprises several sequence-related and functionally diverse neuropeptides: molt-inhibiting hormone (MIH), vitellogenesis-inhibiting hormone (VIH), MOIH, and several CHH subtypes^15–17^. The peptides of the CHH superfamily members consist of 72 to at least 80 amino acids, and share six cysteine residues that form three intramolecular disulfide bridges^18–22^.

The snow crab is giant deep-sea predator in the benthic ecosystems of the northern hemisphere. Unlike females, the terminal molt is not required for the sexual maturation of males because spermatogenesis takes place in male snow crabs and in spider crabs prior to the terminal molt^23,24^. Sexually-matured males are classified into two stages: the adolescent stage, in which spermatogenesis is observed despite the small carapace size, and the mature stage, which is a mature individual bearing a large carapace. The molting between adolescence and young adulthood, and between young adulthood and the complete adult are referred to as the adolescent molt and terminal molt, respectively^25^. Individuals that have undergone a terminal molt are incapable of additional molting^2,25^. Male adolescent *C. opilio* continues to molt proportionately to its previous size until the terminal molt at which time an allometric increase in chela size occurs^2,26^. In addition to chela enlargement, behavioral patterns change for breeding. In fact, in competition between a male after the terminal molt and a larger adolescent male, the former was more likely to dispossess his rival or prevent a takeover^27^.

From an aquaculture perspective, the snow crab is a highly important commercial fisheries resource. Indeed, the mature male snow crab is a commercially important resource in the North Pacific and Northwest Atlantic^28^, and adult males with enlarged chela (after the terminal molt) are more expensive to trade than adolescent males in Japan. Considering its commercial importance, we tried to understand the molecular basis of the terminal molt in the snow crab. We investigated the circulating MF concentrations before or after the terminal molt in the male snow crab using liquid chromatography/mass spectrometry (LC–MS) and confirmed that its concentration varies obviously after the terminal molt, as in previous studies. Then, we generated de novo transcriptome assemblies from the eyestalk ganglion of males from before and after the terminal molt to obtain molecular insight into the regulation of physiological changes in males after the terminal molt.

## Results

### Methyl farnesoate titer in the male hemolymph

**Figure 1:**
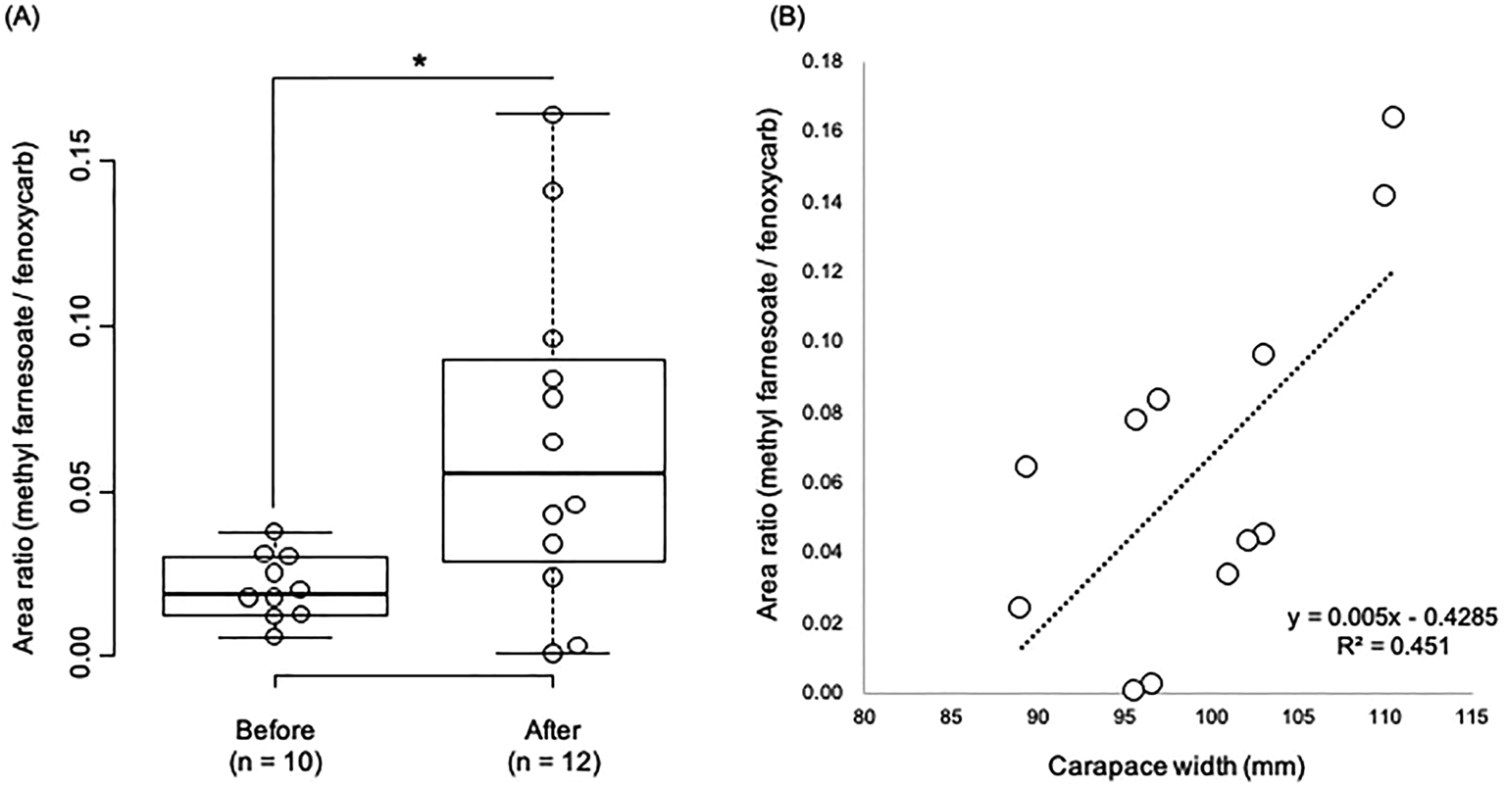
Area ratios compared between methyl farnesoate (MF) and fenoxycarb (internal control) exhibited MF levels in the hemolymph of snow crabs before or after the terminal molt (n = 10 and 12, respectively) (**A**). The asterisk indicates a significant difference between groups (Welch’s* t*-test, p < 0.01). The relationship between MF levels and carapace width in snow crabs after the terminal molt showed a positive correlation (**B**).

Circulating MF levels were significantly higher after the terminal molt (Fig. 1A). However, the level of MF in half of the individuals after the terminal molt was not significantly different from that of individuals before the terminal molt. We thus examined whether there was a correlation between body size and innate MF levels of individuals after the terminal molt. A positive correlation trend between them was suggested (Fig. 1B).

### De novo transcriptome assembly

Using the total RNAs extracted from the eyestalk ganglion, Illumina Nova Seq 6000 sequencing yielded a total of 100,879,987 paired-end clean reads (DDBJ Sequence Read Archive [DRA] accession number DRA014112). The de novo transcriptome assembly produced 60,078 putative transcripts using Trinity, Evidentialgene, and Corset pipelines. Among them, 23,678 transcripts had significant BLAST similarity hits with publicly available protein sequences. The final transcripts were evaluated by BUSCO for completeness based on expectations of gene content against the eukaryotic gene dataset. The completeness result is provided in Table 1.

**Table 1:** Transcript completeness from BUSCO analysis.

BUSCO evaluation metrics	Result
Complete BUSCOs (C)	247 (96.9%)
Complete and single-copy BUSCOs (S)	216 (84.7)
Complete and duplicated BUSCOs (D)	31 (12.2)
Fragmented BUSCOs (F)	4 (1.6%)
Missing BUSCOs (M)	4 (1.5%)
Total BUSCO groups searched	255

### Identification of MF-, sinus gland-, and biogenic amine-related differentially expressed transcripts

**Figure 2:**
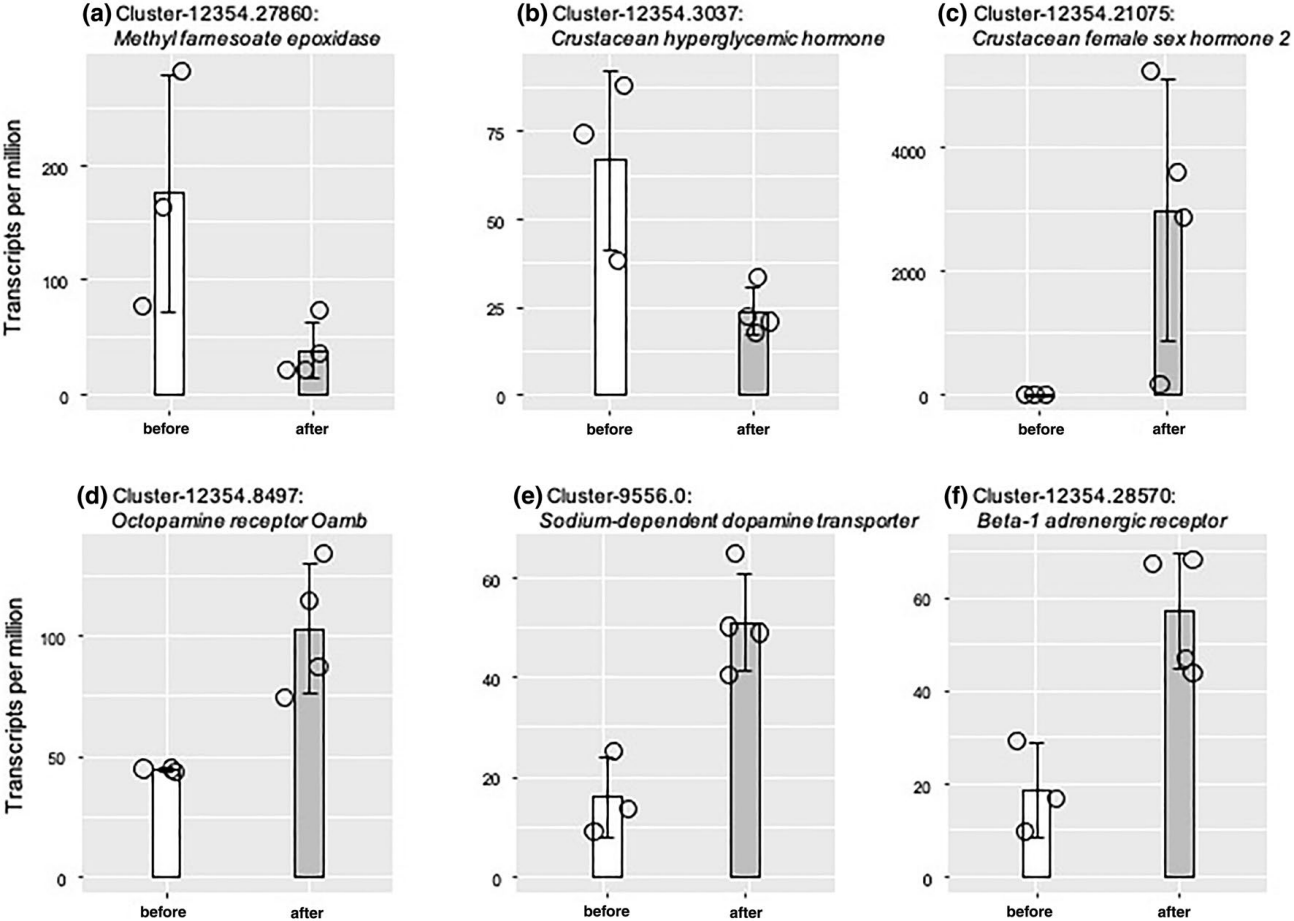
Expression profiles of before or after terminal molt-biased differentially expressed transcripts. Each circle indicates a biological replicate, and bars indicate the standard deviation. Refer to Supplemental Table S2 for a complete listing of differentially expressed transcripts.

**Figure 3:**
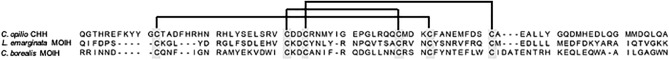
Alignment of Cluster-12354.3037 with the MOIH of other decapods. The six conserved cysteine residues are shown in a gray box. Disulfide bridges are indicated by lines. Accession numbers are as follows: MOIHs of *Libinia emarginata* (AF144660.1) and *Cancer borealis* (QKO41654).

Transcriptomic profiles clearly separated samples before and after the terminal molt (Fig. S4A). Among the 60,078 constructed transcripts, 291 and 332 transcripts were screened as differentially expressed transcripts (DETs; false discovery rate < 0.05) in individuals before and after the terminal molt, respectively (Fig. S4B and Table S1). GO enrichment analysis identified 11 and 23 GO terms in individuals before and after the terminal molt, respectively (Tables S2). Enriched GO terms associated with biological processes after the terminal molt consisted of several metabolic processes such as “regulation of nitrogen compound metabolic process (GO:0051171)”, “regulation of primary metabolic process (GO:0080090)”, “nucleic acid phosphodiester bond hydrolysis (GO:0090305)”, and “mRNA catabolic process (GO:0006402)” (Table S2), implying that the metabolic state of the eyestalk ganglion changes markedly before and after the terminal molt. At first, we searched for transcripts encoding MF-biosynthesis, degradation, and downstream pathways in DETs, and found a transcript encoding *methyl farnesoate epoxidase* that converts MF into JHIII in insects (the active JH form is in insects but not in decapod crustaceans) (Table S3). The expression pattern of *methyl farnesoate epoxidase* was significantly lower after the terminal molt (Fig. 2a). Although sinus gland-derived peptides are known to regulate various biological processes, not enough has been done to analyze the physiological functions of snow crabs. Therefore, we next searched for transcripts encoding sinus gland-derived peptides in DETs (Table S3). As a result, two transcripts encoding *crustacean hyperglycemic hormone* (*CHH*) and *crustacean female sex hormone 2* (*CFSH2*) were found. The expression level of *CHH* was significantly lower after the terminal molt (Fig. 2b) whereas that of *CFSH2* was dramatically higher after the terminal molt (Fig. 2c). A previous study found that the CHH of *L. emarginata* had dual effects and may function both as CHH (control of blood glucose level) and as a MOIH, which is known to negatively regulate MF synthesis^29^. Based on the MF titers (Fig. 1A) and *CHH* expression pattern (Fig. 2b), it was hypothesized that this molecule (Cluster-12354.3037) may act as a MOIH in *C. opilio*. Therefore, we conducted alignment analysis and found that this molecule has high similarity with other MOIH sequences that contain six conserved cysteine residues (Fig. 3).

According to a previous study on social insects, the soldier caste exhibits aggressive behavior due to increased circulating concentrations of juvenile hormones and the consequent activation of biogenic amine pathways^30^. Based on this knowledge, we hypothesized that increased circulating MF levels activate the biogenic amine pathways, which are responsible for the more aggressive behavior of male snow crabs during inter-male competition for successful mating with females after the terminal molt. Therefore, we searched for biogenic amine-related factors in DETs (Table S3) and identified three transcripts that encode *Octopamine receptor Oamb* (Fig. 2d), *Sodium-dependent dopamine transporter* (Fig. 2e), and *Beta-1 adrenergic receptor* (Fig. 2f), whose expression patterns were consistently up-regulated after the terminal molt.

## Discussion

This study combined measurement of circulating MF levels and an assessment of the transcriptome of the eyestalk ganglion of the male snow crab before and after the terminal molt to investigate the physiological mechanisms underlying alterations in behavioral patterns before and after the terminal molt. Our LC–MS results indicated that mean MF titers in males after the terminal molt were significantly higher than those before the terminal molt, which is highly consistent with previous studies in the spider crab that reported higher levels of MF in adults compared to adolescents^8,31^. Similarly, we attempted to make comparisons with a previous study on snow crab^10^. Our sampled snow crabs corresponded to the "old-shell" before and after the terminal molt in the previous study^10^. In that study, the MF concentrations of both "old-shell" groups tended to be higher after the terminal molt, although there was no statistical difference^10^, which is consistent with our results. Moreover, the positive correlation that we found between body length and MF titers in terminal molting individuals suggests that circulating MF levels may control several traits. Previous studies demonstrated that larger individuals of the terminal molt have higher reproductive capacity and higher MF titers^4,8^, and that a positive correlation between high levels of MF and reproductive behavior existed^8,9^. Our data also suggest that larger terminal molting males may be responsible for higher reproductive success in snow crab, and MF might make this possible. The individual differences in MF titers, even among terminal molting individuals, may be related to the number of years since the last molt. It has been suggested that eyestalk and gastric mill ossicles (stomach teeth) can be used to estimate age in several decapod crustaceans, although these traits cannot be used to estimate age in the snow crab after the terminal molt^32^. In the future, if an appropriate age estimation method can be developed, it will be possible to examine the correlation between the time elapsed since the terminal molt and the circulating MF levels.

The transcriptome of the eyestalk ganglion demonstrated that lower expression levels of *methyl farnesoate epoxidase*, a member of *cyp15*, which converts MF to JH III in insects, suggesting that it might act in the primal step of MF metabolism in decapod crustaceans^33–35^. Moreover, as a terminal molt-biased transcript, a *CHH* (Cluster-12354.3037) was identified. CHHs are known to act as multifunctional peptides. For example, CHHs of the penaeid shrimp may have dual functions, hyperglycemic and vitellogenesis-inhibiting activities, because its vitellogenesis is inhibited by CHH molecules purified from the sinus glands of the kuruma prawn *Marsupenaeus japonicus*^36^. Additionally, several recent studies reported new physiological roles of CHH, for example, osmoregulation^37^ and the metabolism of carbohydrates^38^, ammonia^39^, amino acids, and nucleotides^40^. Indeed, the MOIH that was identified in the spider crab showed two functions, mandibular organ inhibiting, and hyperglycemic effects^13^. Based on the alignment of amino acid sequences of the snow crab CHH and other known MOIHs, we found that the snow crab CHH shared high similarity to MOIHs of other decapods with six well-conserved cysteine residues^29,41^, suggesting that the snow crab CHH may act as a MOIH. Therefore, an obvious decrease in its expression pattern after the terminal molt might activate MF synthesis. Originally, eyestalk ablation was used in classical endocrinological studies to investigate the role of specific eyestalk-derived hormones. Our study demonstrated that an increase in MF titers after the terminal molt without eyestalk ablation resulted in the suppression of expression of genes encoding MOIH and MF-degrading enzymes.

In decapod crustaceans, the insulin-like androgenic factor (IAG) and the crustacean female sex hormone (CFSH) are the two main endocrine factors involved in the development of sexually dimorphic characteristics^42,43^. Unlike IAG, CFSH has been isolated as a sinus gland-derived peptide from the eyestalk of the female blue crab, *Callinectes sapidus*. Originally, it was shown that CFSH plays a central role in the development of female secondary sex characteristics, including the development of reproductive traits, by silencing *CFSH* and/or bilateral eyestalk ablation^43^. It is now known that CFSH is widely conserved in decapod crustaceans and, such as in blue crabs, is involved in the development of secondary sexual characteristics in females of the mud crab, *Scylla paramamosain*^44^. Moreover, in some cases, it was found that CFSH is not female-specific, as in *S. paramamosain*^45^, the kuruma prawn *Marsupenaeus japonicus*^46^, and the giant freshwater prawn, *Macrobrachium rosenbergii*^47^. In our study, we found that the expression of CFSH was dramatically increased in the eyestalk ganglion of male snow crabs after the terminal molt, whereas it remained at a baseline expression level in males before the terminal molt. Our preliminary experiment showed that such an increase in CFSH gene expression was not observed in female snow crabs after the terminal molt (data not shown). These findings indicate that the snow crab CFSH has a unique function to promote male characteristics such as behavioral changes. In fact, the physiological functions of CFSH are still largely unknown. Although it was reported that prepubertal *S. paramamosain* males showed a high expression level of CFSH, its expression was extremely reduced in the mature stage^45^. Additionally, its ovarian isoform was found in the kuruma prawn, suggesting its involvement in some reproductive processes^48^. Based on this knowledge, it is suggested that the function of CFSH is not only female-specific but is also diversified among decapods. A future expression analysis in the female snow crab is expected to elucidate the function of CFSH in this species.

Our transcriptome using the eyestalk ganglion revealed that expression levels of three biogenic amine-related factors, namely the octopamine receptor, a dopamine transporter, and adrenergic receptor-related transcripts, were up-regulated in males after the terminal molt. Studies of social insects provide clues to this mystery. In many insects, biogenic amines (e.g., serotonin, dopamine, and octopamine) are involved in locomotion as neuromodulators^30^. Especially in eusocial insects such as ants, bees, and termites, their biogenic amines are strongly linked with several social behaviors via the modulation of physiological states^49^. Various studies have demonstrated that soldiers’ brains contain higher levels of tyramine and octopamine than those of workers in the damp-wood termite *Hodotermopsis sjostedti*^50^, that the dopamine level is associated with the locomotor activity of workers in the termite *Zootermopsis nevadensis*^51^, that octopamine regulates the division of labor in the honey bee *Apis mellifera*^52,53^, and that biogenic amines such as serotonin, dopamine, and octopamine in the brain of the queen ant, *Formica japonica*, change with early colony formation^54^. Moreover, many studies using several bee species have demonstrated that biogenic amines such as octopamine and dopamine interact with juvenile hormones to regulate locomotor behavior^55,56^, division of labor^52,53^, and reproduction^57^. Taken together, juvenile hormones and biogenic amines are concertedly involved in behavioral changes associated with castes and aging in social insects. Although a few studies demonstrated that several biogenic amines trigger behavioral changes in some decapod species^58,59^, less is known about the relationship between biogenic amines and hemolymphatic MF levels. Future studies of the interactions of biogenic amines and MF in decapod crustaceans will provide insight into the molecular underpinnings of behavioral changes in male snow crabs.

In this study, circulating MF levels were measured by LC–MS, and the transcriptome of the eyestalk ganglion of the male snow crab *C. opilio* before or after the terminal molt was conducted to estimate the physiological mechanisms altering behavioral patterns after the terminal molt. Our data indicate that the increase in MF titers after the terminal molt may be caused by suppression of the genes encoding MF-degrading enzymes and MOIH. Moreover, the eyestalk ganglion transcriptome revealed that behavioral changes after the terminal molt may be driven by the activation of biogenic amines (e.g., octopamine and dopamine). Thus, although a further study that tests the potential effects of MF and biogenic amines is necessary, the MF-biogenic amines system might modulate the reproductive behavior of male snow crabs. Our findings have the potential to be applied to aquaculture. If an artificially method to control puberty molt and terminal molt is developed, it would be possible to induce the terminal molt in livestock husbandry of snow crabs.

## Materials and methods

### Ethical statement

No specific permissions were required for our studies, which did not involve endangered or protected invertebrate species. All efforts were made to minimize the suffering of the animals.

### Animals

In November 2020, male snow crabs before or after the terminal molt were purchased from Echizen Town Fisheries Cooperative Association in Fukui Prefecture, Japan. Ten healthy (no missing limbs) males before the terminal molt (carapace width: 98.21 ± 6.50 mm, wet weight: 335.4 ± 67.9 g) and twelve males after the terminal molt (carapace width: 100.0 ± 7.01 mm, wet weight: 388.7 ± 81.8 g) were used for both quantification of MF and RNAseq analysis (Table S4). To judge whether an individual was before or after the terminal molt, the carapace width and chela height of each crab was measured to the nearest 0.1 mm using digital calipers according to a previous study^2^. The discriminant values to judge whether crabs were before or after the terminal molt was calculated using the following formula:

$$ {\text{Y }} = {-}\;{16}.{\text{5221 ln}}\left( {{\text{CW}}} \right) \, + { 14}.{5}00{\text{3 ln}}\left( {{\text{CH}}} \right) \, + { 33}.{8949} $$

Values of CW and CH are the carapace width (mm) and chela length (mm), respectively. If Y values were < – 0.01123, than the individual was estimated as being before the terminal molt^25^. All data relating to the carapace width and chela height (Fig. S1) is listed in Table S4.

The shell condition of snow crabs was generally defined as "new-shell" (within one year after molting) and "old-shell" (more than one year after molting), judged by the cleanness of the carapace, according to previous studies^10,60^, but criteria such as abrasions and epibiotic growth on the carapace were tuned for the North American population, implying that these definitions do not apply to our study. Instead, we estimated that all snow crabs used in this study were categorized as "old-shell", based on previous criteria because the molting period of snow crabs nearing maturity in the Sea of Japan is mainly from September to October^25^. In this study, all crabs were caught in November, so if the crabs had molted in September or October, the shell should have been clean and soft-shelled. However, as is shown in Fig. S2, there were many epibiotic organisms on the carapace^61^, so we concluded that we were using individuals that had been molting for more than one year.

### Quantification of methyl farnesoate

Three hundred micro liter of hemolymph samples were removed from the base of the chela and homogenized in 300 µL of 100% methanol with 1 pmol of fenoxycarb (Sigma-Aldrich, St. Louis, MO, USA) as an internal standard. Ten and 12 replicates (each replicate derived from a single snow crab) were prepared before and after the terminal molt (Table S4). Each sample was transferred into a glass vial, and 100 µL of 2% NaCl was added. They were extracted three times with 300 µL of hexane (Wako, Osaka, Japan). After adding hexane, the sample was vortexed vigorously, incubated for 5 min at room temperature, and centrifuged (900 × g) for 5 min. The hexane (upper) phase was collected in a new glass vial. The combined hexane extract (900 µL) was dried completely using a N_2_ gas spray and dissolved in 20 µL of acetonitrile (Wako). The sample was stored at − 20 °C until analysis.

To quantify the MF titers in snow crabs, LC–MS was used as follows. First, 2 µL from each 20 µL of extracted sample was separated on a PFP column (Nucleodur PFP 3 μm, 1.0 × 100 mm) using a gradient elution of water with 0.1% formic acid (mobile phase A)/methanol with 0.1% formic acid (mobile phase B) (0 min 30% B, 3 min 90% B, 25 min 90% B) at a flow rate of 30 µL/min, using an Eksigent microLC 200 system (AB SCIEX, Framingham, MA, USA). MF was detected with a TripleTOF 5600 system (AB Sciex). The mass spectrometer was operated under ESI positive mode ionization with Multiple Reaction Monitoring (MRM). MRM transitions included *m/z* from 251.2 to 191.18 for MF (declustering potential [V]: 90, collision energy [V]: 30), and *m/z* from 302.3 to 256.1 for fenoxycarb (declustering potential [V]: 30, collision energy [V]: 25), and source parameters were: curtain gas, 25 psi/spray; voltage, 5500 V; temperature, 550 °C; ion source gas 1, 25 psi; ion source gas 2, 50 psi. To evaluate the significant differences of MF titers before and after the terminal molt, Welch’s *t*-test was performed.

### Preparation of tissue samples for RNAseq

Three healthy males before the terminal molt (carapace width: 95.11 ± 2.49 mm, wet weight: 303.6 ± 17.3 g) and four males after the terminal molt (carapace width: 107.8 ± 2.78 mm, wet weight: 481.9 ± 23.5 g) were used for RNAseq analysis (Table S4). Both sides of the eyestalks were cut, and then each eyestalk ganglion, including the sinus gland, was extracted (Fig. S3). Each sample (a total of 14 samples consisting of seven left- or right-eyestalks) was stored separately in RNAlater reagent (Thermo Fisher Scientific, city, MA, USA) until use. Total RNA was extracted using ISOGEN II (Nippon Gene Co Ltd., Tokyo, Japan) and the RNeasy Micro Kit (Qiagen, CA, USA) from the left-side eyestalk ganglion, following the manufacturers’ protocols. The quality and concentration of total RNA were validated by NanoDrop (Thermo Fisher Scientific) and 2100 Bioanalyzer (Agilent Technologies, city, CA, USA). All left-side eyestalk ganglion samples were used to develop the de novo transcriptome.

### Construction of cDNA libraries and sequencing

The cDNA libraries were constructed by a Novogene service (Gene Nex, Tokyo, Japan). In brief, mRNA from snow crabs was enriched using oligo(dT) beads. For long-non-coding libraries, rRNA was removed using the Ribo-Zero kit that isolated the mRNA. First, the mRNA was fragmented randomly by adding fragmentation buffer, then the cDNA was synthesized by using an mRNA template and random hexamer primers, after which a custom second-strand synthesis buffer (Illumina), dNTPs, RNase H, and DNA polymerase I were added to initiate second-strand synthesis. Second, after a series of terminal repairs, A ligation, and sequencing adaptor ligation, the double-stranded cDNA library was completed through size selection and PCR enrichment. Then, all libraries were sequenced using an Illumina NovaSeq 6000 platform that was equipped with a 150 bp paired-end module.

### Data analysis pipeline

The quality of output sequences was inspected using the Fast QC program (version 0.11.2, available online at: http://www.bioinformatics.babraham.ac.uk/projects/fastqc). All reads of each group (males before the terminal molt: three replicates, and males after the terminal molt: four replicates) were co-assembled using the RNAseq de novo assembler Trinity (version 2.9.1) in the paired-end mode^62^. Then, assembled transcriptomes of both males were merged as a single fasta file that was submitted to the EvidentialGene tr2aacds pipeline (available online at: https://sourceforge.net/projects/evidentialgene/) to generate a single assembly with minimal redundancy while maximizing the maintenance of long coding sequence regions in each contig. Finally, the tr2aacds pipeline produced an “okay” set of transcripts that were regarded as the optimal and representative transcript datasets that were used as a reference transcriptome in this study. The reads from each biological replicate were mapped to the assembled transcripts for quantification by Salmon (version 1.1.0) with the “--dumpEq” option^63^. Then, contigs were clustered based on the proportion of shared reads and expression by Corset (version 1.09)^64^. Corset generated the clusters and outputs as a table of counts containing the number of reads uniquely assigned to each cluster. The completeness of orthologs of the Corset-generated transcriptome was examined using BUSCO version 5.0.0 against eukaryote_odb10 (Creation date: 2020-09-10, number of species: 70, number of BUSCOs: 255).

By using the Corset-generated count data, differentially expressed genes were calculated using the DESeq2 package in the SARTools package (version 1.6.6)^65^ between males before and after the terminal molt. Using BLASTX (threshold E-values = 1E−03) with the AC-DIAMOND package (version 2.0.4.142)^66^, Corset-generated transcripts were aligned with the NCBI protein database NR (non-redundant). Gene Ontology (GO) annotation was completed using BLAST2GO in OmixBox software version 2.0.10^67^. Multiple alignments were constructed using ClustalX 2.1 with default settings^68^.

## Supplementary Information

Supplementary Table S1.

Supplementary Table S2.

Supplementary Table S3.

Supplementary Table S4.

Supplementary Figures.

## Data Availability

Sequencing data have been deposited in DDBJ under the accession code DRA014112.

## References

[1] Song Y (2017). Ecdysone receptor agonism leading to lethal molting disruption in Arthropods: Review and adverse outcome pathway development. Environ. Sci. Technol..

[2] Conan GY, Comeau M (1986). Functional maturity and terminal molt of male snow crab, *Chionoecetes opilio*. Can. J. Fish. Aquat. Sci..

[3] Carlisle DB (1957). On the hormonal inhibition of moulting in decapod crustacea. II. The terminal anecdysis in crabs. J. Mar. Biol. Ass. U. K..

[4] Rotllant G (2000). Role of ecdysteroids and methyl farnesoate in morphogenesis and terminal moult in polymorphic males of the spider crab *Libinia emarginata*. Aquaculture.

[5] Tamone SL, Adams MM, Dutton JM (2005). Effect of eyestalk-ablation on circulating ecdysteroids in hemolymph of snow crabs, *Chionoecetes opilio*: Physiological evidence for a terminal molt. Integr. Comp. Biol..

[6] Laufer H (1993). Ecdysteroids and juvenoids in two male morphotypes of *Libinia emarginata*. Insect Biochem. Molec. Biol..

[7] Tamone SL (2007). The relationship between circulating ecdysteroids and chela allometry in male tanner crabs: Evidence for a terminal molt in the genus *Chionoecetes*. J. Crustacean Biol..

[8] Sagi A, Ahl JSB, Danaee H, Laufer H (1994). Methyl farnesoate levels in male spider crabs exhibiting active reproductive behavior. Horm. Behav..

[9] Laufer, H. *et al*. Hormone and reproductive strategies in spider crabs with emphasis on commercially important species. In: *High Latitude Crabs: Biology, Management, and Economics* 383–387 (1996).

[10] Zaleski MAF, Tamone SL (2014). Relationship of molting, gonadosomatic index, and methyl farnesoate in male snow crab (*Chionoecetes opilio*) from the eastern Bering Sea. J. Crustacean Biol..

[11] Laufer H (1987). Identification of a Juvenile hormone-like compound in a crustacean. Science.

[12] Homola E, Sagi A, Laufer H (1991). Relationship of claw form and exoskeleton condition to reproductive system size and methyl farnesoate in the male spider crab, *Libinia emarginata*. Invertebr. Reprod. Dev..

[13] Liu L, Laufer H (1996). Isolation and characterization of sinus gland neuropeptides with both mandibular organ inhibiting and hyperglycemic effects from the spider crab *Libinia emarginata*. Arch. Insect Biochem. Physiol..

[14] Wainwright G (1996). Structure and significance of mandibular organ-inhibiting hormone in the crab, *Cancer pagurus*: Involvement in multihormonal regulation of growth and reproduction. J. Biol. Chem..

[15] Kegel G, Reichwein B, Tensen CP, Keller R (1991). Amino acid sequence of crustacean hyperglycemic hormone (CHH) from the crayfish, *Orconectes limosus*: Emergence of a novel neuropeptide family. Peptides.

[16] Keller R (1992). Crustacean neuropeptides: Structures, functions and comparative aspects. Experientia.

[17] Soyez D (1997). Occurrence and diversity of neuropeptides from the crustacean hyperglycemic hormone family in arthropods. A short review. Ann. N. Y. Acad. Sci..

[18] Chang ES, Prestwich GD, Bruce MJ (1990). Amino acid sequence of a peptide with both molt-inhibiting activity and hyperglycemic activities in the lobster *Homarus americanus*. Biochem. Bioph. Res. Co..

[19] Huberman A, Aguilar MB (1988). Single step purification of two hyperglycaemic neurohormones from the sinus gland of *Procambarus bouvieri*: Comparative peptide mapping by means of high-performance liquid chromatography. J. Chromatogr..

[20] Huberman A (1993). Primary structure of the major isomorph of the crustacean hyperglycemic hormone (CHH-I) from the sinus gland of the Mexican crayfish *Procambarus bouvieri* (Ortmann): Interspecies comparison. Peptides.

[21] Kegel G (1989). Amino acid sequence of the crustacean hyperglycemic hormone (CHH) from the shore crab, *Carcinus maenas*. FEBS Lett..

[22] Soyez D (1990). Neuropeptides from the sinus gland of the lobster *Homarus americanus*: Characterization of hyperglycemic peptides. Gen. Comp. Endocrinol..

[23] Hinsch G (1972). Some factors controlling reproduction in the spider crab, *Libinia emarginata*. Biol. Bull..

[24] Sainte-Marie B, Raymond S, Brethes J-C (1995). Growth and maturation of the benthic stages of male snow crab, *Chionoecetes opilio* (Brachyura: Majidae). Can. J. Fish. Aquat. Sci..

[25] Yamasaki A, Kuwahara A (1991). The terminal molt of male snow crab in the Japan Sea. Nippon Suisan Gakkaishi.

[26] Comeau M, Conan GY (1992). Morphometry and gonad maturity of male snow crab, *Chionoecetes opilio*. Can. J. Fish. Aquat. Sci..

[27] Sainte-Marie B, Sévigny JM, Gauthier Y (1997). Laboratory behavior of adolescent and adult males of the snow crab (*Chionoecetes opilio*) (Brachyura: Majidae) mated noncompetitively and competitively with primiparous females. Can. J. Fish. Aquat. Sci..

[28] Stevens BG, Miller TJ, Lovrich GA, Thiel M (2020). Crab fisheries. Fisheries and Aquaculture, The Natural History of the Crustacea.

[29] Liu L, Laufer H, Wang Y, Hayes T (1997). A neurohormone regulating both methyl farnesoate synthesis and glucose metabolism in a crustacean. Biochem. Biophys. Res. Commun..

[30] Libersat F, Pflueger HJ (2004). Monoamines and the orchestration of behavior. Bioscience.

[31] Laufer H, Ahl JSB (1995). Mating behavior and methyl farnesoate levels in male morphotypes of the spider crab, *Libinia emarginata* (Leach). J. Exp. Mar. Biol. Ecol..

[32] Rebert AL (2020). Evaluation of a direct age estimation method for terminally molted male snow crab *Chionoecetes opilio* (Fabricius 1788) (Decapoda: Brachyura: Oregoniidae). J. Crustacean Biol..

[33] Miyakawa H (2018). Ecdysteroid and juvenile hormone biosynthesis, receptors and their signaling in the freshwater microcrustacean *Daphnia*. J. Steroid Biochem. Mol. Biol..

[34] Toyota K (2022). Juvenile hormone synthesis and signaling disruption triggering male offspring induction and population decline in cladocerans (water flea): Review and adverse outcome pathway development. Aquat. Toxicol..

[35] Tu S (2022). Molecular characterization of the cytochrome P450 epoxidase (CYP15) in the swimming crab *Portunus trituberculatus* and its putative roles in methyl farnesoate metabolism. Biol. Bull..

[36] Khayat M (1998). Hyperglycaemic hormones inhibit protein and mRNA synthesis in in vitro-incubated ovarian fragments of the marine shrimp *Penaeus semisulcatus*. Gen. Comp. Endocrinol..

[37] Sun D (2019). Crustacean hyperglycemic hormone of *Portunus trituberculatus*: Evidence of alternative splicing and potential roles in osmoregulation. Cell Stress Chaperones.

[38] Liu A (2019). A novel crustacean hyperglycemic hormone (CHH) from the mud crab *Scylla paramamosain* regulating carbohydrate metabolism. Comp. Biochem. Physiol. A Mol. Integr. Physiol..

[39] Zhang X (2020). Crustacean hyperglycemic hormone (CHH) regulates the ammonia excretion and metabolism in white shrimp, *Litopenaeus vannamei* under ammonia-N stress. Sci. Total Environ..

[40] Li W, Chiu K-H, Lee C-Y (2019). Regulation of amino acid and nucleotide metabolism by crustacean hyperglycemic hormone in the muscle and hepatopancreas of the crayfish *Procambarus clarkii*. PLoS ONE.

[41] Wiwegweaw A, Udomkit A, Panyim S (2004). Molecular structure and organization of crustacean hyperglycemic hormone genes of *Penaeus monodon*. J. Biochem. Mol. Biol..

[42] Levy T, Sagi A (2020). The, “IAG-switch”—A key controlling element in decapod crustacean sex differentiation. Front. Endocrinol..

[43] Zmora N, Chung JS (2014). A novel hormone is required for the development of reproductive phenotypes in adult female crabs. Endocrinology.

[44] Jiang Q (2020). Role of crustacean female sex hormone (CFSH) in sex differentiation in early juvenile mud crabs, *Scylla paramamosain*. Gen. Comp. Endocrinol..

[45] Liu A (2018). Crustacean female sex hormone from the mud crab *Scylla paramamosain* is highly expressed in prepubertal males and inhibits the development of androgenic gland. Front. Physiol..

[46] Kotaka S, Ohira T (2018). cDNA cloning and in situ localization of a crustacean female sex hormone-like molecule in the kuruma prawn *Marsupenaeus japonicus*. Fish. Sci..

[47] Thongbuakaew T (2019). Identification and characterization of a crustacean female sex hormone in the giant freshwater prawn, *Macrobrachium rosenbergii*. Aquaculture.

[48] Tsutsui N, Kotaka S, Ohira T, Sakamoto T (2018). Characterization of distinct ovarian isoform of crustacean female sex hormone in the kuruma prawn *Marsupenaeus japonicus*. Comp. Biochem. Physiol. Part A Mol. Integr..

[49] Kamhi JF, Arganda S, Moreau CS, Traniello J (2017). Origins of aminergic regulation of behavior in complex insect social systems. Front. Syst. Neurosci..

[50] Ishikawa Y, Aonuma H, Sasaki K, Miura T (2016). Tyraminergic and octopaminergic modulation of defensive behavior in termite soldier. PLoS ONE.

[51] Yaguchi H, Inoue T, Sasaki K, Maekawa K (2016). Dopamine regulates termite soldier differentiation through trophallactic behaviours. R. Soc. Open Sci..

[52] Schulz DJ, Barron AB, Robinson GE (2002). A role for octopamine in honey bee division of labor. Brain Behav. Evol..

[53] Schulz DJ, Sullivan JP, Robinson GE (2002). Juvenile hormone and octopamine in the regulation of division of labor in honey bee colonies. Horm. Behav..

[54] Aonuma H, Watanabe T (2012). Changes in the content of brain biogenic amine associated with early colony establishment in the queen of the ant, *Formica japonica*. PLoS ONE.

[55] Bloch G, Meshi A (2007). Influences of octopamine and juvenile hormone on locomotor behavior and period gene expression in the honeybee, *Apis mellifera*. J. Comp. Physiol. A.

[56] Sasaki K, Nagao T (2013). Juvenile hormone–dopamine systems for the promotion of flight activity in males of the large carpenter bee *Xylocopa appendiculata*. Naturwissenschaften.

[57] Harano K, Sasaki K, Nagao T, Sasaki M (2008). Influence of age and juvenile hormone on brain dopamine level in male honeybee (*Apis mellifera*): Association with reproductive maturation. J. Insect Physiol..

[58] Huber R (1997). Serotonin and aggressive motivation in crustaceans: Altering the decision to retreat. Proc. Natl. Acad. Sci. USA.

[59] Livingstone MS, Harris-Warrick RM, Kravitz EA (1980). Serotonin and octopamine produce opposite postures in lobsters. Science.

[60] Jadamec, L. S., Donaldson, W. E. & Cullenberg, P. Biological field techniques for *Chionoecetes crabs*. In University of Alaska Sea Grant. AK- SG-99-02, University of Alaska, Fairbanks, AK (1999).

[61] Nagasawa K, Fujiwara K (2008). Two piscicolid leeches (Hirudinida) and their cocoons on snow crabs *Chionoecetes opilio* in Japan, with the first record of *Johanssonia arctica* from the Sea of Japan. Biogegraphy.

[62] Grabherr MG (2011). Trinity: Reconstructing a full-length transcriptome without a genome from RNA-seq data. Nat. Biothechnol..

[63] Patro R (2017). Salmon provides fast and bias-aware quantification of transcript expression. Nat. Methods.

[64] Davidson NM, Oshlack A (2014). Corset: Enabling differential gene expression analysis for *de novo* assembled transcriptome. Genome Biol..

[65] Varet H (2016). SARTools: A DESeq2- and EdgeR-based R pipeline for comprehensive differential analysis of RNA-seq data. PLoS ONE.

[66] Mai H (2018). AC-DIAMOND v1: Accelerating large-scale DNA-protein alignment. Bioinformatics.

[67] Götz S (2008). High-throughput functional annotation and data mining with the Blast2GO suite. Nucleic Acids Res..

[68] Larkin MA (2007). Clustal W and Clustal X version 2.0. Bioinformatics.

