# Supplementary material for: Eyestalk transcriptome and methyl farnesoate titers provide insight into the physiological changes in the male snow crab, Chionoecetes opilio, after its terminal molt
Source: Sci Rep. 2023 May 3;13:7204. doi: 10.1038/s41598-023-34159-y (PMC10156855; doi:10.1038/s41598-023-34159-y)
Supplement: Supplementary file 5 — Supplementary Figures. [file 41598_2023_34159_MOESM5_ESM.docx]

Eyestalk transcriptome and methyl farnesoate titers provide insight into the physiological changes in the male snow crab, *Chionoecetes opilio*, after its terminal molt

Kenji Toyota^1,2,3*^, Takeo Yamamoto^4^, Tomoko Mori^5^, Miyuki Mekuchi^6^, Shinichi Miyagawa^2^, Masaru Ihara^7^, Shuji Shigenobu^5^, Tsuyoshi Ohira^3*^

^1^Noto Marine Laboratory, Institute of Nature and Environmental Technology, Kanazawa University, Ogi, Noto-cho, Ishikawa 927-0553, Japan.

^2^Department of Biological Science and Technology, Faculty of Advanced Engineering, Tokyo University of Science, 6-3-1 Niijuku, Katsushika-ku, Tokyo 125-8585, Japan.

^3^Department of Biological Sciences, Faculty of Science, Kanagawa University, 2946 Tsuchiya, Hiratsuka, Kanagawa, 259-1293, Japan.

^4^Miyazu Field Station, Japan Fisheries Research and Education Agency, Miyazu, Kyoto, 626-0052, Japan.

^5^Trans-Omics Facility, National Institute for Basic Biology, Okazaki 444-8585, Japan.

^6^Yokohama Field Station, Fisheries Resources Institute, Japan Fisheries Research and Education Agency, 2-12-4 Hukuura, Kanazawa-ku, Yokohama, Kanagawa, 236-8648, Japan.

^7^Faculty of Agriculture and Marine Science, Kochi University, 200 Monobe-Otsu, Nankoku, Kochi 783-8502, Japan.

*** Correspondences:**Dr. Kenji Toyota
toyotak@se.kanazawa-u.ac.jp

Prof. Tsuyoshi Ohira
ohirat-bio@kanagawa-u.ac.jp

**Supplementary figures**


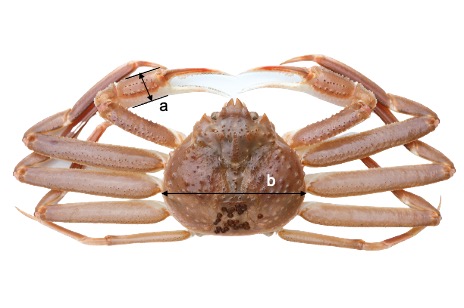


Figure S1

Male snow crab showing the measured part of chela height (A) and carapace width (B).


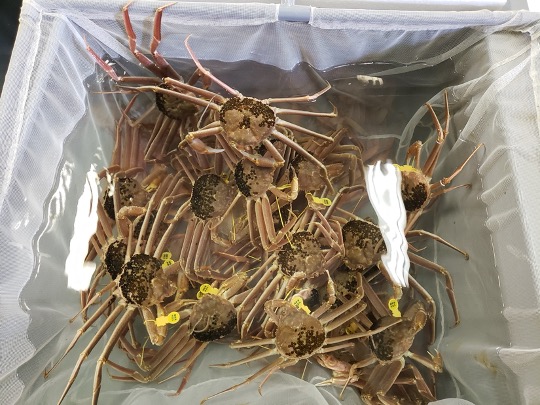


Figure S2

Snapshot of snow crabs used in this study.


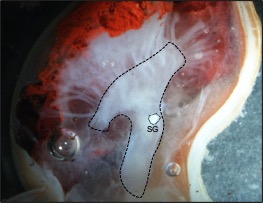


Figure S3

Internal anatomy of the eyestalk of the snow crab. The dotted line indicates the dissected area from which total RNA was extracted. SG: sinus gland.


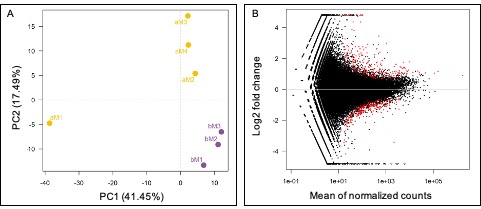


Figure S4

Principal component analysis of our RNAseq (A). "aM" and "bM" indicate the male after or before the terminal molt, respectively. MA plots of the differentially expressed transcripts between snow crabs before and after the terminal molt (B). Red spots indicate transcripts that were significantly different between both groups.
